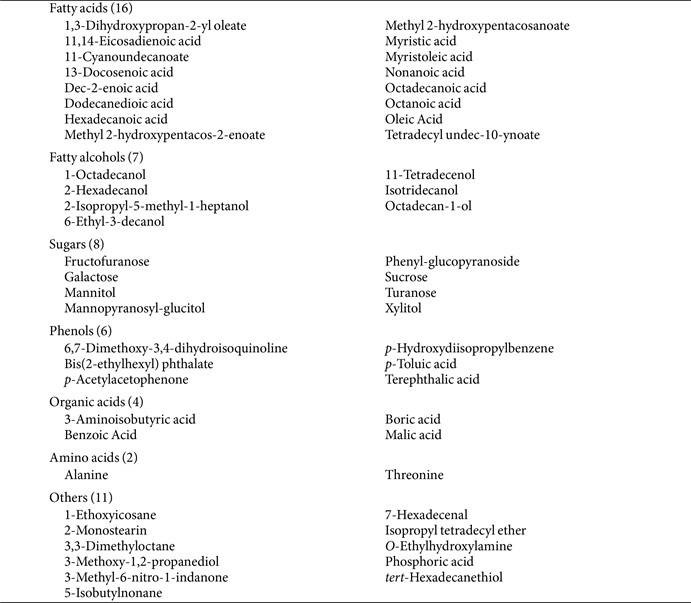# Profiling of Metabolite Changes in Lettuce Leaves during Fermentation by *Bacillus subtilis*

**DOI:** 10.4014/jmb.2501.01026

**Published:** 2025-05-15

**Authors:** Dong Young Lee, Yu Rim Yun, Byeong-Jun Ji, Sewol Park, Subin Kim, Sun-Hee Lee, Hyun-Soo Chun, Eun Ju Yun

**Affiliations:** 1 Department of Biotechnology, The Catholic University of Korea, Bucheon 14662, Republic of Korea; 2 HumanEnos LLC., Wanju 55347, Republic of Korea; 3 Department of Biomedical Sciences and Institute for Medical Science, Jeonbuk National University Medical School, Jeonju 54907, Republic of Korea; 4 Division of Biotechnology, Jeonbuk National University, Iksan 54596, Republic of Korea; 5 Department of Biotechnology, Graduate School, Korea University, Seoul 02841, Republic of Korea

**Keywords:** Lettuce, *Bacillus subtilis*, whole-cell bioconversion, fermentation, metabolite profiling, gas chromatography and mass spectrometry

## Abstract

Metabolic profiling is a valuable tool for elucidating the biochemical pathways and key metabolites involved in the health benefits associated with microbial fermentation. In this study, we investigated the metabolic changes occurring during the fermentation of lettuce leaves by *Bacillus subtilis*, a widely studied bacterium known for its diverse metabolic capabilities. Through non-targeted metabolic profiling, we identified and characterized metabolites that may contribute to the beneficial effects of fermented lettuce. Using gas chromatography-mass spectrometry (GC/MS), we identified 54 metabolites in the fermented lettuce samples. Additionally, we elucidated the alterations in metabolite profiles during the bioconversion of lettuce using *B. subtilis*. Notably, 11,14-eicosadienoic acid, 13-docosenoic acid, and oleic acid were either produced or enriched during bioconversion, were identified as potential contributors to the enhanced nutritional and bioactive properties of fermented lettuce. This study underscores the potential of metabolic profiling to uncover the metabolic pathways and specific metabolites associated with health benefits in fermented foods. These findings pave the way for developing functional foods with improved nutritional value and bioactivity.

## Introduction

Lettuce (*Lactuca sativa* L.), an annual leafy vegetable belonging to the family *Asteraceae*, is a widely consumed food known for its numerous health benefits, including anti-inflammatory, cholesterol-lowering, and antidiabetic properties [1]. Owing to these health benefits, lettuce extract is frequently used as an active ingredient in functional foods and nutraceuticals [2, 3]. The primary bioactive components of lettuce that provide biological activity are carotenoids and phenolic compounds, such as dicaffeoyl tartaric acid, β-carotene, and lutein [4, 5]. Furthermore, many functional compounds in lettuce exist in flavonoid glucoside forms, including luteolin-7-*O*-glucuronide, kaempferol 3-malonylglucoside, and quercetin-3-*O*-glycoside [6].

Bioconversion or fermentation of raw food resources, such as soybean germ extract and lettuce extract, using Generally Recognized as Safe (GRAS)-grade food microbes is widely employed to enhance physiological activities [7, 8]. For instance, soybean germ extract is rich in isoflavones, a class of phytoestrogen such as genistein and glycitein, that structurally resemble 17-β-estradiol and can mimic estrogenic activity by binding to estrogen receptors [9, 10]. However, these isoflavones predominantly exist in glycoside forms, such as genistin, daidzin, and glycitin, along with their acetyl and malonyl conjugates [11]. Due to their higher polarity and molecular weight compared to their aglycone forms (*e.g.*, genistein and glycitein), the physiological activities of the glycosidic isoflavones are considerably limited [12]. A recent study demonstrated that the addition of the probiotic *Lactobacillus gasseri* to soybean germ extract remarkably enhances its anti-menopausal effects in an ovariectomized rat model compared to untreated soybean germ extract [13]. This improvement is attributed to the bioconversion of isoflavone glycosides in the soybean germ extract into their aglycone forms, thereby increasing their physiological activity. Similarly, the health benefits of fermented lettuce extract using *Bacillus subtilis* have been reported, including its ability to induce immune responses and inhibit the migration and proliferation of human fibroblast-like synoviocytes [14, 15].

*B. subtilis* is a type of microorganism that is widely utilized in food fermentation due to its ability to produce a broad spectrum of hydrolytic enzymes, including amylases and proteases [16, 17]. This bacterium synthesizes extracellular polysaccharides, polypeptides, and lipopeptides that serve as bioactive compounds [18-20]. Particularly, certain strains of *B. subtilis* exhibit antimicrobial activity against various pathogens, including *Vibrio parahaemolyticus*, *Vibrio alginolyticus*, and *Staphylococcus aureus* [21-23]. In soil environments, *B. subtilis* contributes to plant growth by enhancing nitrogen fixation and producing siderophores that suppress the proliferation of harmful pathogens [24-26].

Given that the bioactive phytochemicals in raw food resources, such as polyphenols, carotenoids, and chlorophyll, are typically classified as secondary metabolites, their analysis has primarily been conducted using liquid chromatography–mass spectrometry (LC/MS) [27, 28]. In contrast, gas chromatography-mass spectrometry (GC/MS)-based global metabolite profiling that predominantly targets primary metabolites during the bioconversion process, remains relatively underexplored. Since fermentation or bioconversion often involves the breakdown of compounds through catabolic pathways or degradation, leading to the formation of low molecular weight compounds, analyzing these smaller metabolites is critically important. Representative examples of low-molecular-weight metabolites include specific fatty acids, such as short-chain fatty acids (SCFAs) and oleic acid. These metabolites have attracted considerable attention due to their physiological activity [29-32].

In this study, we investigated the bioconversion of lettuce leaves using *B. subtilis* and monitored changes in metabolite profiles through GC/MS analysis. Multivariate statistical analysis (*i.e.*, principal component analysis) and hierarchical cluster analysis were performed to identify potential metabolites contributing to the health benefits of fermented lettuce extract. These findings provide valuable insights into the metabolic changes that occur during the bioconversion of lettuce extracts by *B. subtilis*.

## Materials and Methods

### Fermentation of Lettuce Leaves Using *B. subtilis*

The bioconversion of lettuce leaves through microbial fermentation using *B. subtilis* KCTC12501BP was conducted by Human Enos (Republic of Korea). *B. subtilis* was seed-cultured in a 3-L baffled flask containing 500 ml of medium composed of 3 g/l peptone, 3 g/l yeast extract, 3 g/l malt extract, and 20 g/l sucrose at 37°C with agitation at 150 rpm for 24 h. The culture was then transferred to a 500-L fermentor containing 360 L of medium with 10–20 g/l sucrose, 5–8 g/l soy peptone, 5 g/l soluble starch, 1–2 g/l dipotassium phosphate, 0.2–0.5 g/l magnesium sulfate, 5 g/l yeast extract, 0.1 g/l manganese sulfate, and 0.05–0.1 g/l calcium chloride, incubated at 37°C with agitation at 120–150 rpm for 24 h.

Briefly, ground lettuce was mixed with distilled water in a ratio of 1:1, followed by the addition of approximately 1% (1.0 × 10^8^ CFU/ml) of *B. subtilis*. The bioconversion was performed at 30°C for 21 days under optimized aeration, temperature, and pH conditions. Under optimized conditions, the chemical oxygen demand was maintained between 350 and 450 mg/l at the end of fermentation, with the pH ranging from 7 to 8.

Throughout the bioconversion process, samples were meticulously collected at intervals of 0, 8, and 15 days for metabolic profiling, with 40 ml of each sample collected each time point. The supernatant was then obtained by centrifugation (1.5 ton/h, disc separator; Alfa Tech Korea Corp., Republic of Korea).

### Sample Preparation for GC/MS Analysis

Prior to GC/MS analysis, 50 μl of each samples were vacuum-dried for 20 min using a centrifugal vacuum concentrator (Operon, Republic of Korea) equipped with an ultra-low-temperature cold trap bath (JSR, Republic of Korea). For derivatization, methoxyamination and trimethylsilylation reactions were carried out as follows: First, 10 μl of 40 mg/ml methoxyamine hydrochloride in pyridine (Sigma-Aldrich, USA) was added to the dried sample and incubated at 30°C with shaking at 1,000 rpm for 90 min. Subsequently, 45 μl of *N*-methyl-*N*-trimethylsilyl trifluoroacetamide (MSTFA, Sigma-Aldrich) was added and incubated at 37°C with shaking at 1,000 rpm for 30 min.

### GC/MS Analysis

Following derivatization, the samples were analyzed using a GC/MS system (GC-2030/MS QP2020, Shimadzu GCMS-QP Series, Shimadzu, Japan) equipped with an SH-I-5Sil MS Cap column (30 m × 0.25 mm, 0.25 μm film thickness; Shimadzu). For metabolite analysis, 1 μl of the derivatized sample was injected into the gas chromatography (GC) in split mode at a ratio of 5. The GC oven temperature was initially set to 50°C for 1 min, and then increased at a rate of 15°C/min to 280°C, where it was maintained for 10 min. Mass spectra were recorded within the scan range of 50-550 m/z, with the ion source and interface temperatures set at 250°C and 280°C, respectively. The fermentation process was conducted on an industrial scale with a working volume of 10 tons in a 25-ton tank. Due to the scale of fermentation, GC/MS analysis was performed in analytical triplicate, in which samples were collected and aliquoted into three separate Eppendorf tubes for sample preparation.

### Data Processing and Statistical Analysis

The raw data obtained from the GC/MS analysis was processed using the Automated Mass Spectral Deconvolution and Identification System (AMDIS) software for peak detection and deconvolution. Mass spectral similarity was employed as a criterion for peak identification [33]. The processed data was then uploaded to SpectConnect for peak alignment, and a data matrix was generated using the Golm Metabolome Database (GMD) mass spectral reference library (http://gmd.mpimp-golm.mpg.de/) [34].

Multivariate statistical analyses, including principal component analysis (PCA), were conducted using Statistica software (version 7.1; StatSoft, USA). Additionally, hierarchical cluster analysis (HCA), visualized as a heat map, was performed in R using the Pheatmap R package (RStudio, v2023.12.1; Posit Software, PBC).

## Results and Discussion

### GC/MS-Based Metabolite Profiling of Fermented Lettuce Leaves

To compare the metabolite profiles during the fermentation of lettuce leaves with *B. subtilis* KCTC12501BP, sampling was conducted on days 0, 8, and 15. In total, 54 metabolites were identified through GC/MS analysis and categorized into five groups: fatty acids and their derivatives, sugars and their derivatives, amino acids, phenols, and acids (Table 1). Among these, fatty acids and their derivatives were the most abundant category (Table 1).

**Fig. 1:**
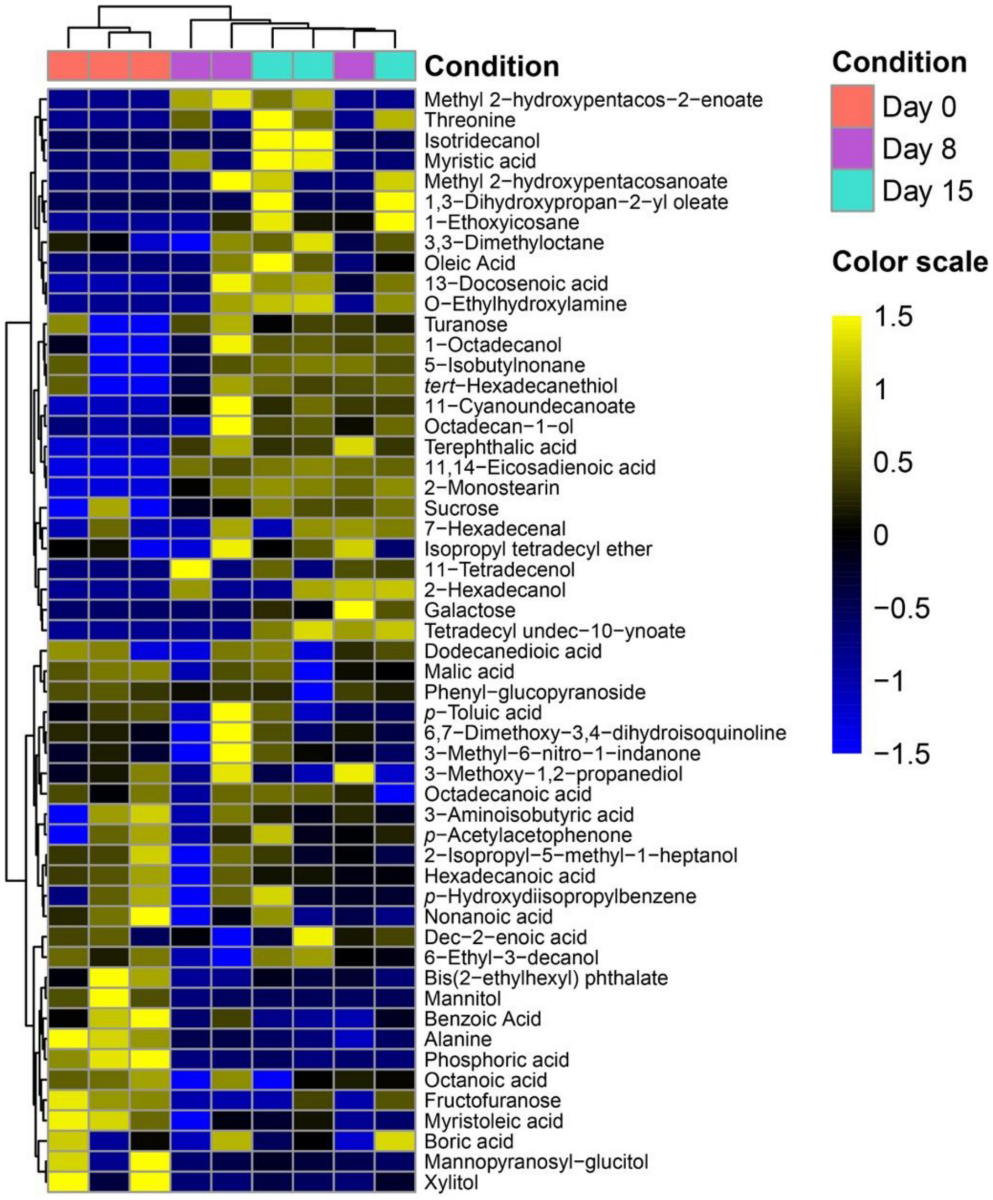
Heat map of 54 metabolites identified from the fermented lettuce leaves using *B. subtilis*. The x-axis represents three different time points, days 0, 8, and 15, and y-axis represents the 54 metabolites identified in this study. All experiments were performed in analytical triplicates. The color scale on the right side of the figure represents the range of z-scores.

The identified metabolites were then subjected to HCA, as visualized in the heat map (Fig. 1). The results revealed distinct clustering of metabolite profiles before and after fermentation, with day 0 forming a separate cluster compared to days 8 and 15. The abundance of most identified metabolites in this study increased on days 8 and 15 relative to day 0, except for myristoleic acid, mannitol, alanine, and phosphoric acid (Fig. 1). The observed increase in metabolite levels during fermentation (*i.e.*, days 8 and 15) is likely attributed to the activation of catabolic pathways in *B. subtilis*, that facilitated the breakdown of macromolecules in lettuce leaves into smaller components, as detected by GC/MS analysis [35].

### Notable Metabolites Changed during Fermentation

**Fig. 2:**
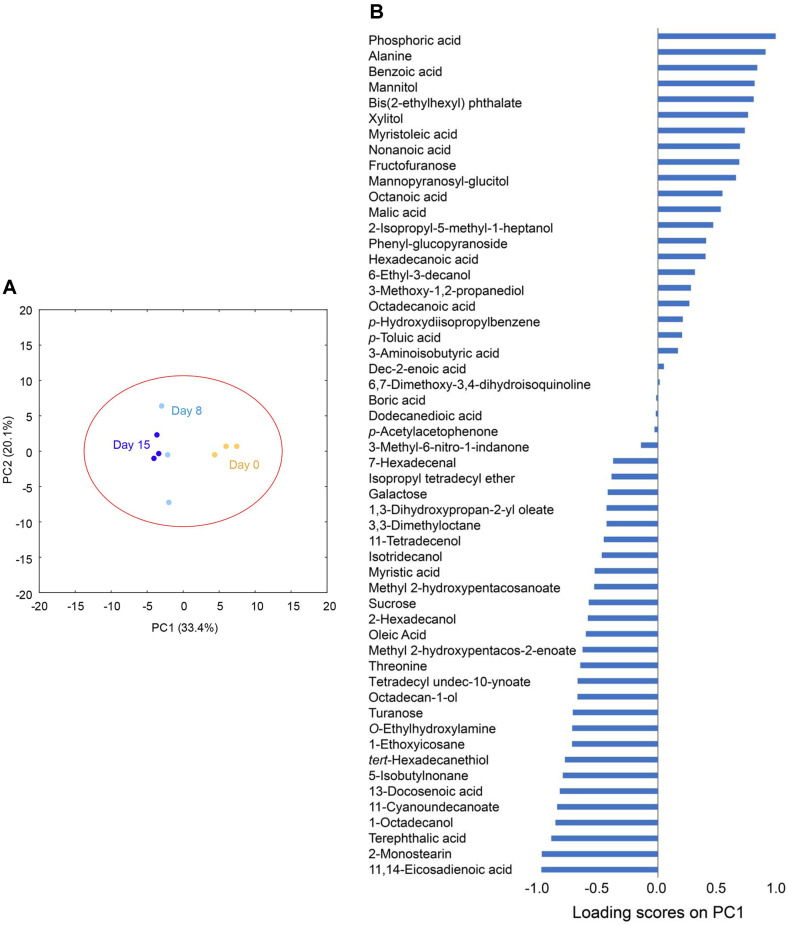
(A) Principal component analysis (PCA) scatter plot of 54 metabolites from fermented lettuce leaves using *B. subtilis*. The labels in the PCA scatter plot represent the three different sampling time points, days 0, 8, and 15. (**B**) The loading values of 54 identified metabolites on PC1. All experiments were performed in analytical triplicates.

To investigate differences in the metabolite profiles across fermentation days and identify metabolites that changed significantly during the bioconversion process, PCA was performed (Fig. 2A). The PCA results showed clear separation between fermentation days (*i.e.*, 0, 8, and 15 days), with an *R^2^X* value of 0.535, indicating a good fit for the PCA model. *R^2^X* is the cumulative variance contribution rate, which indicates how much of the total variance in the dataset is explained by the selected components. In metabolomics analysis, an *R^2^X* value above 0.4 is generally considered to indicate a sufficient level of variance explanation by the model [36, 37]. Changes in the metabolite profiles were evident during bioconversion, with PC1 primarily contributing to the separation of metabolite profiles across fermentation days (Fig. 2A).

The total of 54 metabolites with loading scores on PC1 revealed that phosphoric acid, alanine, benzoic acid, and mannitol contributed most positively to PC1 (Fig. 2B). This indicates that the abundances of these metabolites acquired by GC/MS were highest on 0 day of fermentation, and decreased during the fermentation process, likely due to bioconversion or consumption by B. subtillis. Conversely, fatty acids and fatty alcohols, such as 11,14-eicosadienoic acid, 2-monosterin, and 1-octadecanol, were the most negative contributors to PC1. This suggests their abundance increased during fermentation, likely as a result of bioconversion (Fig. 2B). The observed increases in 11,14-eicosadienoic acid, 2-monostearin, and 1-octadecanol during the fermentation of lettuce leaves with *B. subtilis* are likely associated with lipid metabolism and the degradation of cell membrane components [38]. The enzymatic activity of *B. subtilis*, including lipases and esterases, may have facilitated the release and transformation of fatty acids and glycerides from lettuce cell membrane.

Sugars such as glucose and sucrose also negatively contributed to PC1 (Fig. 2B) and were probably produced during the bioconversion process. This could be attributed to the cleavage of glycosidic bonds in flavonoid glucosides, such as kaempferol-3-malonylglucoside, and quercetin-3-*O*-glycoside or their release from plant lettuce cells because sucrose is one of the major sugars synthesized in lettuce [6, 39].

### Potential Bioactive Metabolites in Fermented Lettuce Leaves

**Fig. 3:**
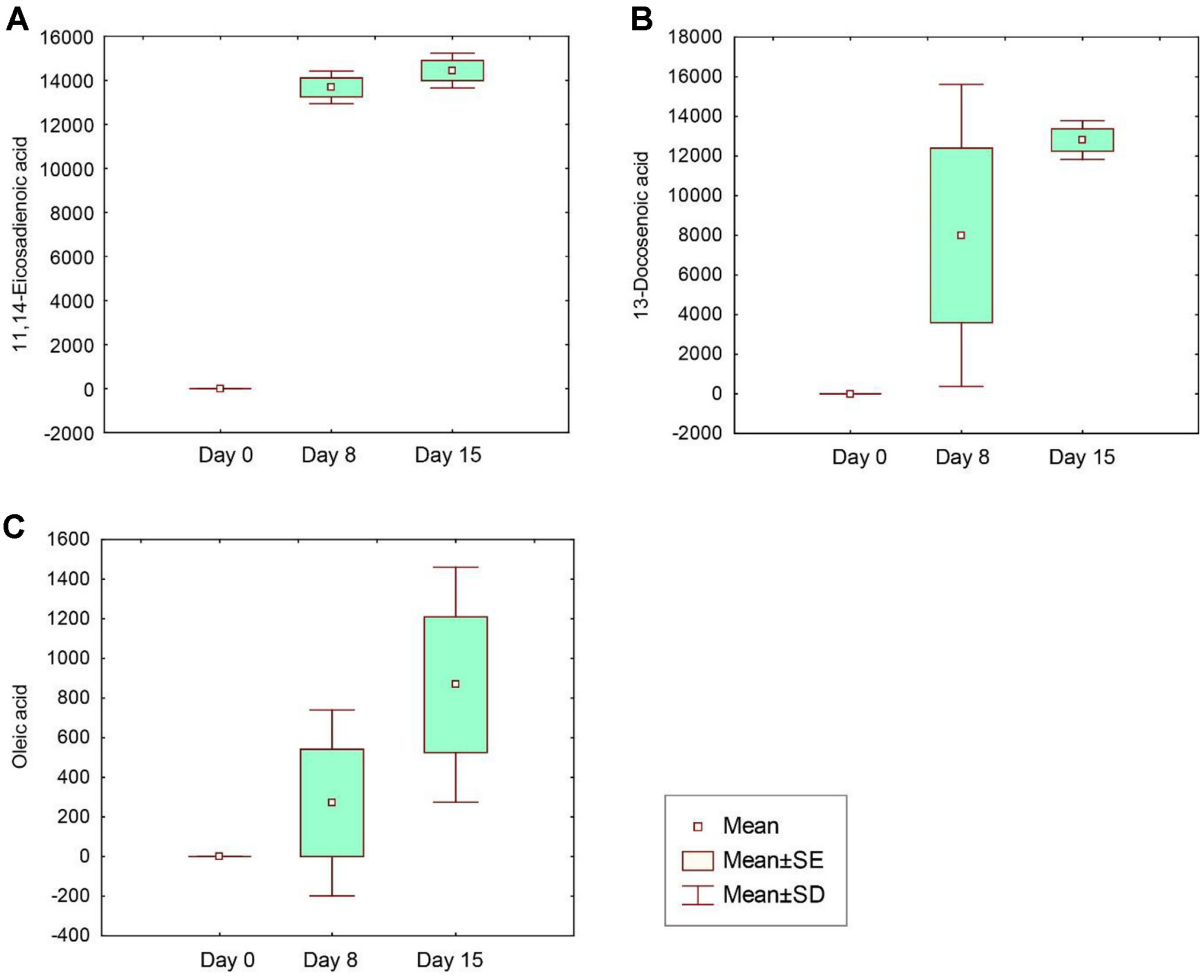
Base peak heights of (A) 11,14-eicosadienoic acid, (B) 13-docosenoic acid, and (C) oleic acid, depicting a significant increase during the bioconversion of lettuce leaves using *B. subtilis*, as presented in the box-whisker plots. The y-axis values represent the height of the base peak, with m/z 67, 117, and 339 used for 11,14- eicosadienoic acid, 13-docosenoic acid, and oleic acid, respectively. The x-axis labels represent the three different sampling time points: days 0, 8, and 15. All experiments were performed in analytical triplicates.

Possible metabolites produced during the bioconversion process that may confer beneficial effects were identified (Fig. 3). Among the various unsaturated fatty acids detected in the fermentation products, 11,14-eicosadienoic acid and 13-docosenoic acid exhibited the most significant increase (Figs. 3A and 3B). First, 11,14-eicosadienoic acid, an omega-6 polyunsaturated fatty acid, was specifically investigated for its potential health benefits. A previous study indicated that it possesses anti-inflammatory properties by modulating the production of pro-inflammatory cytokines and mediators in various cell types, particularly immune cells such as macrophages [40]. Its anti-inflammatory activity may also contribute to its reported anticancer effects [41]. Prior to bioconversion, 11,14-eicosadienoic acid was not detected, but was produced during the bioconversion on days 8 and 15 (Fig. 3A), likely as a result of enzymatic activity from *B. subtilis*.

Similarly, 13-docosenoic acid and oleic acid, showing similarity to 11,14-eicosadienoic acid, were also produced during fermentation (Figs. 3B and 3C). Both 13-docosenoic acid and oleic acid are monounsaturated omega-9 fatty acids. Notably, 13-docosenoic acid, a 22-carbon monounsaturated fatty acid, can be metabolized into oleic acid *in vivo*. *Bacillus* species are capable of converting 13-docosenoic acid to oleic acid via a process involving fatty acid elongation and desaturation [42]. This conversion may include β-oxidation of fatty acids to shorten the carbon chain, followed by the activity of fatty acid desaturases that introduce a necessary double bond at the appropriate position.

Oleic acid has significant applications in the pharmaceutical and health industries, where it is utilized as an excipient in pharmaceutical formulations and as an emulsifying or solubilizing agent in aerosol products [43]. Furthermore, oleic acid has been proposed to slow the progression of adrenoleukodystrophy, a fatal neurodegenerative disorder affecting the brain and adrenal glands, and may possess cognition-enhancing properties [44]. Oleic acid is believed to contribute to the blood pressure-lowering effect of olive oil [45].

The specific fatty acids 11,14-eicosadienoic acid, 13-docosenoic acid, and oleic acid increased during the fermentation process. Future studies should focus on the quantitative analysis of these metabolites using a sample preparation method optimized for target metabolites to determine their absolute concentrations, as their concentration range may be critical for bioactivity and potential physiological functions.

## Conclusion

In conclusion, the bioconversion of raw food materials using GRAS-grade food microorganisms has been widely employed to enhance the physiological activities of active ingredients. In this study, we monitored changes in metabolite profiles during the bioconversion of lettuce using *B. subtilis*. Comprehensive metabolic analysis using GC/MS demonstrated significant alterations in global metabolite profiles throughout the fermentation process. Particularly, the unsaturated fatty acids 11,14-eicosadienoic acid and oleic acid, known for their high physiological activities, including anti-inflammatory and hypotensive (or blood pressure-reducing) effects, were produced during the fermentation process. This study highlights the impact of metabolic profiling in identifying specific metabolites with health benefits in fermented food products. These findings can be leveraged to develop functional foods with enhanced nutritional values and bioactivity, offering promising applications in the food industry.

## Figures and Tables

**Table 1:** A total of 54 metabolites identified in this study.